# Microscopic Live Electrooptic Imaging

**DOI:** 10.1038/s41598-017-08442-8

**Published:** 2017-08-11

**Authors:** Masahiro Tsuchiya, Shinji Fukui, Muneo Yorinaga

**Affiliations:** 1 National Institute of Information and Communications Technology, 4-2-1 Nukui-Kitamachi, Koganei-shi, Tokyo 184-8795 Japan; 2 SOKEN, INC., 14 Iwaya, Hasumi-cho, Nishio-shi, Aichi 445-0012 Japan

## Abstract

Live electrooptic imaging (LEI) in the microscopic range has been successfully demonstrated for the first time. The finest resolution achieved in the present study is 2.7 μm, which is finer than the previous record by more than an order of magnitude. This drastic improvement in the resolution record has been achieved through comprehensive improvement of the limiting factors of the conventional LEI system. Residual limiting factors in the improved system have been systematically analyzed and ideas for even finer resolution have also been presented.

## Introduction

**Figure 1:**
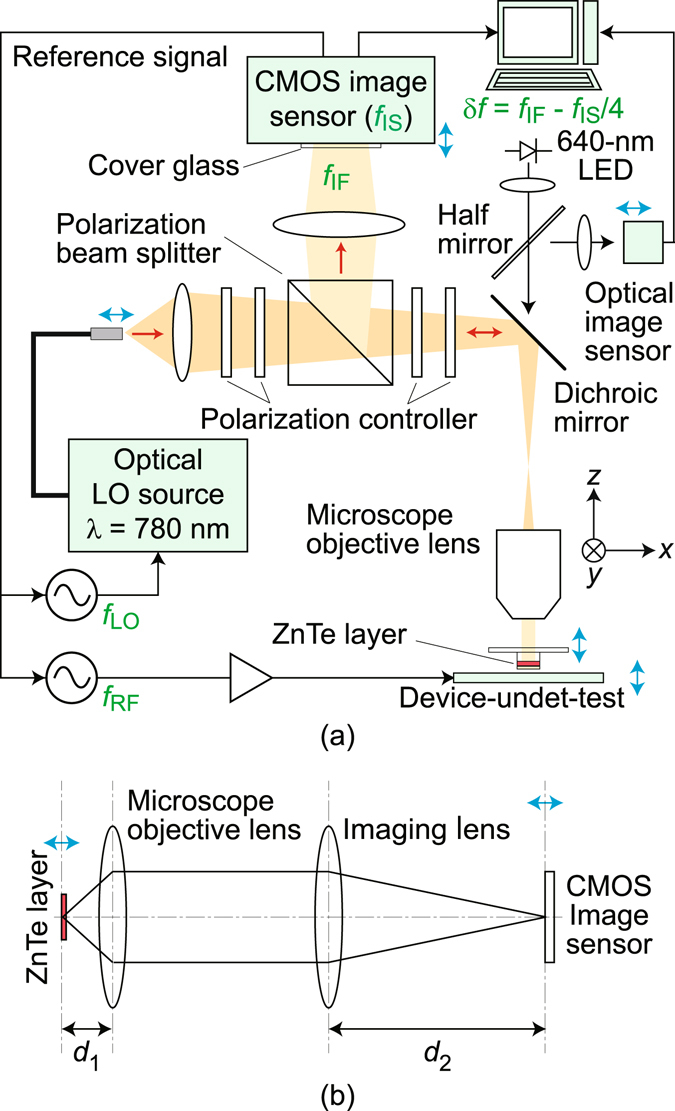
A schematic of the setup for microscopic live electrooptic imaging is shown in (**a**) together with a conceptual drawing of its imaging optics for microscopy in (**b**). Red arrows in (**a**) show 780 nm laser light propagation while blue arrows indicate fine positioners. CMOS: complementary-metal-oxide-semiconductor, *d*_1_ & *d*_2_: focal lengths, IF: intermediate frequency, IS: image sensor, λ: wavelength, LED: light-emitting-diode, LO: local oscillator, RF: radio frequency.

It should be highly attractive if the space-domain behaviors of radio wave, electrical signals and/or noises in and around circuits, as well as conditions of electromagnetic interferences, could be grasped agilely. Prompt visualization of invisible electric fields in terms of both their spatial distributions and dynamics would be the most effective. For this purpose, in addition to various numerical approaches, there is a technique named live electrooptic imaging (LEI)^1–3^ schematically shown in Fig. 1(a), which is unique due to its experimental agility; high frequency electric fields are visualized in real time in the phase-evolving video formats. The LEI technique, whose operation principle is briefly described in the Methods section, is based on two excellent benefits of photonics^4^, ultra-parallel^5, 6^ and ultra-fast^7, 8^ properties, which are merged in an electrooptic (EO) sensor^9, 10^ shaped in the form of a thin plate^11, 12^.

Although further applications are expected in various areas in the future, the following basics have been demonstrated so far: wave vector mapping^13^ and its applications for high-definition wave imaging and waveguide mode analysis^14^, visualization of rotating electric field vectors and their elliptical nature^15^, visualization and decomposition of Bloch states in metamaterial structures^16^, visualization of propagating waves in and around electromagnetic absorbers^17^, and packet imaging^18^. The scheme of detached EO imaging (DEI)^19^ has been added to this list recently. The present status of the LEI technique is indicated by its highest frequency of visualized waves, largest vision area, highest optical magnification ratio, and maximum pixel number, which are 100 GHz^2^, 25 mm square^3^, 8.5^2^, and 256 × 256 = 65,536^20^, respectively.

**Figure 2:**
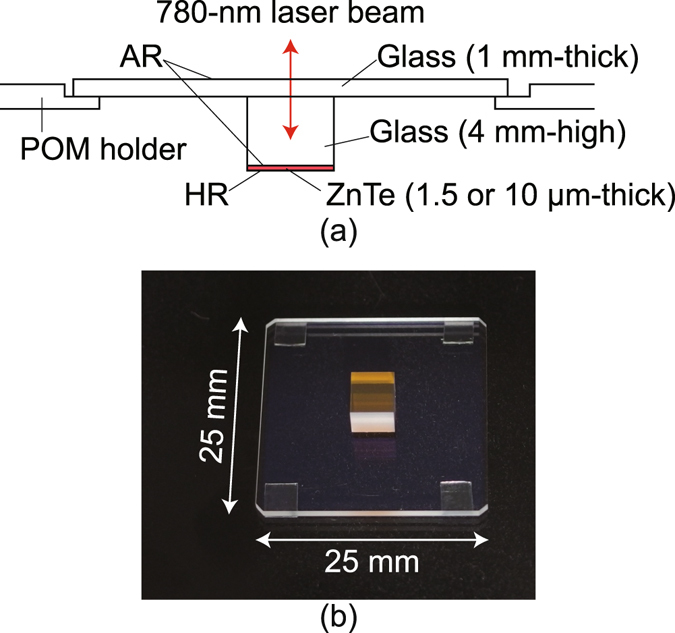
A ZnTe electrooptic sensor plate used in the present study is shown in a cross-sectional side view with its polyoxymethylene (POM) holder schematically (**a**) and photographically with its position upside down (**b**). The ZnTe area is 4.7 mm × 5.2 mm. AR/HR: anti-/high- reflection coated.

In addition to these figures, the highest spatial resolution has been reported as 30 to 40 μm in our recent paper^21^, which was an improvement from the conventional in the sub-millimeter range achieved primarily by reducing the thickness of the ZnTe EO layer on the same glass platform as shown in Fig. 2 to 10 μm and having its proximity contact to circuit patterns. This improvement is reasonable since it is generally supposed that a thinner EO sensor plate leads to a higher spatial resolution at expense of sensitivity, if a fringe-shaped electric field distribution from a circuit pattern is dealt with. The resolution is almost fine enough for patterns on printed circuit boards (PCBs), but is insufficient for microscopic circuit patterns on semiconductor substrates. For this reason, the remaining factors limiting the resolution have been discussed and concluded to be the following: three optics originating factors (restricted diffraction limit, deviated focus, and inadequately large pixel size on EO images) and one EO interaction property (room to thin the ZnTe layer)^21^.

For a finer spatial resolution in a new LEI system, each of these factors has been improved as follows. A microscope objective lens (Fig. 1(a)) was introduced together with an infinity correction optical system (Fig. 1(b)) with fine focus adjusting mechanisms. The magnification power of the optics was tuned precisely, and the ZnTe layer was thinned further to 1.5 μm (Fig. 2(a)). Their details are described in the Method section. This paper will report on the demonstration of a new scheme, microscopic LEI (mLEI), achieved with these modifications for resolution improvement. Discussions aimed at further resolution improvement will also be presented in the Discussion section.

## Results

### Preliminary characterization of the new optics

To characterize the improved optics shown in Fig. 1 preliminarily, optical imaging experiments were conducted on a commercially-available standard resolution target, a 1951USAF glass slide resolution target with positive vacuum-deposited chromium patterns, which was mounted on the same glass platform structure as shown in Fig. 2(a).

Its 640 nm and 780 nm optical images were first acquired from its air side. As illustrated in Fig. 1(a), the 640 nm light-emitting-diode (LED) illumination and an optical image sensor are for optical monitoring of device-under-test (DUT) while the 780 nm laser light illumination and a complementary-metal-oxide-semiconductor (CMOS) image sensor combined with real-time image signal processing are for EO imaging. Their light paths are separated by a dichroic mirror. Clear pattern resolutions at the highest spatial frequency (645.08 mm^−1^) in the target were confirmed in both images, indicating its optical resolution close to the diffraction limit of the microscope objective lens (0.95 μm) as well as sufficient focus adjustability.

The second observation was the target from the reverse side going through the 5 mm thick glass portion, which causes aberration and degrades spatial resolutions in the LEI experiments below. The most detailed of the respective patterns resolved in 640 nm and 780 nm images have spatial frequencies of 456.14 and 322.54 mm^−1^ (corresponding to a resolution around 1.6 μm), and the ratio of these frequencies is 16% less than the wavelength ratio. This deviation could be due to interference fringes observed in the 780 nm laser image, which imply unnecessary residual reflections in the optics. It is thus indicated that the aberration by the glass platform and the interference fringe patterns remain as limiting factors, which degrade the optical resolution from the diffraction limit.

### Optical images of LEI resolution target patterns

**Figure 3:**
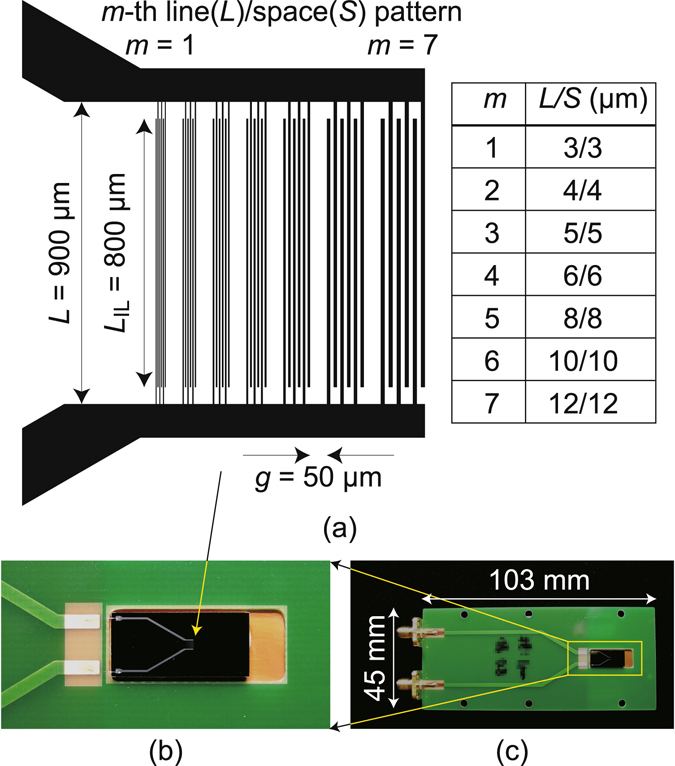
A device-under-test sample with Al interlaced interdigital patterns set as LEI resolution targets is shown. (**a**) is a drawing of the photolithography mask pattern for the Al electrodes with their line and space sizes listed in the table, (**b**) is a region around the Si chip, and (**c**) is a photograph of the overall view. IL: interlaced.

**Figure 4:**
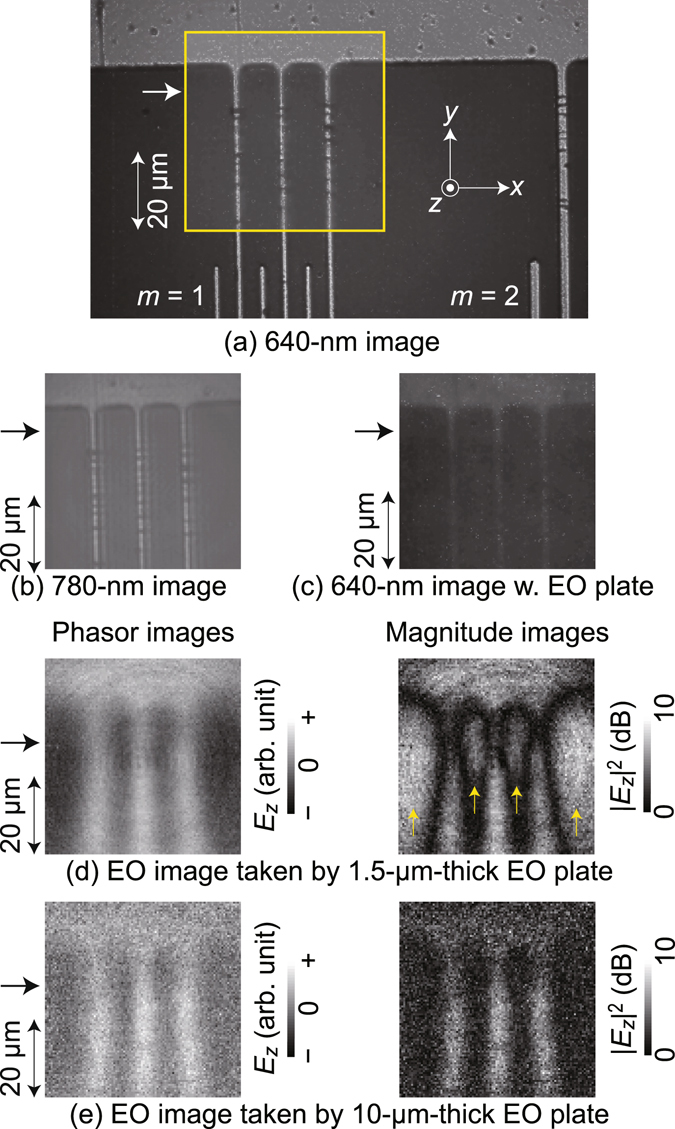
Optical and electrooptic (EO) images of an LEI resolution target pattern of *m* (Fig. 3(a) = 1 are shown. Optical images (**a**) and (**b**) were taken with no EO plate, and image (**c**) was taken with an EO plate with a 1.5 μm thick ZnTe layer. The yellow frame in (**a**) corresponds to the image areas in (**b**) and (**c**). Phasor and magnitude images for the distribution of electric field *E*_*z*_ were taken with EO plates with ZnTe layer thicknesses of 1.5 μm (**d**) and 10 μm (**e**). Heights of the horizontal profiles in Fig. 5 (**a**) are shown by the side arrows. Yellow arrows in (**d**) indicate regions of negative electric fields.

DUT having LEI resolution target patterns on an *n*-type Si substrate, which is shown in Fig. 3, was prepared as mentioned in the Method section. The patterns were optically imaged first with and then without an EO plate beneath the objective lens. Figure 4(a and b) show respective optical images taken with no EO plate for the upper part of the *m* = 1 pattern in Fig. 3(a), whose respective signal-to-noise ratios were evaluated more than 20.9 and 21.6 dB. The linewidth and interval are 1.5 and 10.5 μm, respectively, whose deviation from those in the mask pattern (Fig. 3(a)) is due to the side etching effect. The pattern is resolved well in both, while the image replicas in the right-hand sides of their origins appear in Fig. 4(b). These replicas are ascribed to multiple reflections of the laser light at the CMOS image sensor surface and its cover glass (Fig. 1(a)) as pointed out previously^21^, and could degrade the 780 nm resolution further as another residual limiting factor.

**Figure 5:**
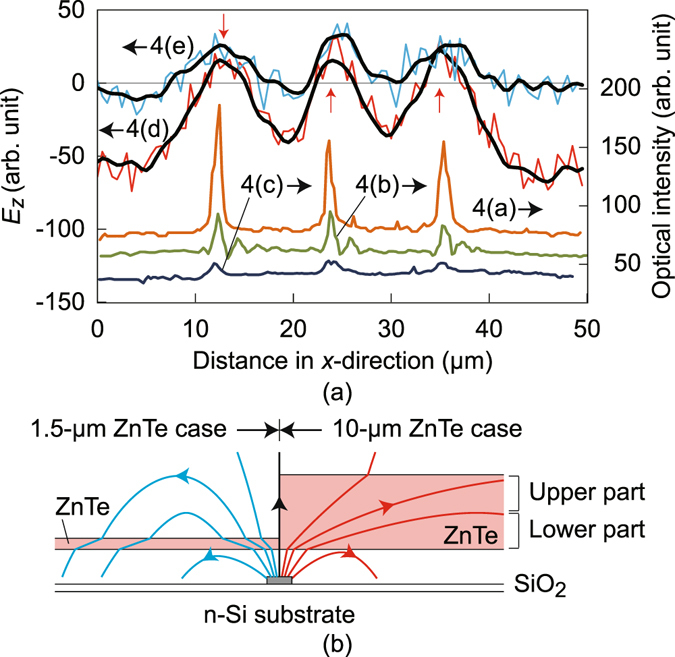
The light-blue and red lines in (**a**) are horizontal profiles of phasor images in Fig. 4(d and e), respectively, whereas those filtered are indicated by black solid lines. Orange, green and dark blue lines are horizontal profiles of the optical images in Fig. 4(a,b and c), respectively. Schematic cross-sectional drawings of electric force lines for the cases of ZnTe layer thickness 1.5 and 10 μm are shown in (**b**), with the goal of assisting in readers’ comprehension.

For quantitative analysis, their line profiles are plotted in the lower part of Fig. 5(a), where the orange and green lines correspond to the horizontal pixel rows indicated by arrows in Fig. 4(a and b), respectively. Their peak widths coincide with the narrowed electrodes while the replicas are clearly recognized as sub-peaks for Fig. 4(b).

A 640 nm image of the Al pattern taken through an EO plate touching the DUT surface is shown in Fig. 4(c), and the appearance is dark due to the low 640 nm round trip transmission of the EO plate (approximately 20%), and also contains electrode images broadened by the above-mentioned aberration. These features are quantitatively indicated by the dark blue line in Fig. 5(a), and the imaging line widths are 2 μm or broader, which gives the optical resolution limit of the present optics for the 1.5 μm wide metal line whereas its future improvement is possible as discussed in the Discussion section.

### Demonstration results of microscopic live electrooptic imaging

LEI experiments sensitive to electric fields in the *z*-direction *E*_z_ have been performed under the same conditions as in Fig. 4(c). The frequency and power of an injected radio frequency (RF) signal were 1.1 GHz and 20 dBm, respectively. Figure 4(d) shows phasor and magnitude images in an 8 bit monochrome gradation, which were acquired using an EO plate with a 1.5 μm thickness ZnTe layer. The former indicates instantaneous *E*_*z*_ fields with the midpoint (128) of the 8 bit gradation scale (256) as the zero level, where the phase was chosen for the maximum contrast. The latter shows squared amplitude in dB, corresponding to power distribution. Apparently, the *E*_*z*_ field from each of the interdigital electrodes is distinguished from the others, and their lateral spread increases downward along the electrode, which can be attributed to the edges of the upward electrodes originating from the lower comb base (Fig. 4(a)). Corresponding images taken with an EO plate with a 10 μm thickness ZnTe layer, which is the same as in our previous publication^21^, are shown in Fig. 4(e). Their mutual comparisons together with individual characteristic features are discussed in the Discussion section.

In order to evaluate the LEI resolution, the phasor image profiles at the 43rd pixel rows shown by the arrows in Fig. 4(d and e) are plotted in the upper part of Fig. 5(a) with light blue and red lines, respectively. Black solid lines are used for the profiles of the images numerically filtered for the sake of noise reduction, where the cutoff wave number is 0.8π μm^−1^. The respective averaged full widths at half maxima of the positive electric field distribution are 2.7 and 4.9 μm for the 1.5 and 10 μm thickness ZnTe layers.

### Summary of the results

Microscopic live electrooptic imaging has been thus successfully demonstrated for the first time. The finest resolution of 2.7 μm was achieved using the 1.5-μm thick ZnTe EO sensor plate, which is finer than the previous record (30 to 40 μm) by more than an order of magnitude. Even with the same 10-μm thick sensor plate as in the previous work, 4.9 μm resolution was achieved, indicating that the optics modification gives rise to the resolution improvement and the residual resolution difference is caused by the different EO plate thicknesses as discussed below. On the other hand, four residual optical factors limiting the resolution were suggested by the optically taken images.

## Discussion

Besides the deviated resolution values, two notable image differences exist between Fig. 4(d and e): (i) the image areas of the negative electric field regions and (ii) the electric field amplitudes just above the line electrodes. Namely, the regions of negative electric fields indicated by yellow arrows in the magnitude image in Fig. 4(d) are faint in Fig. 4(e), and the peak amplitudes shown by red arrows in Fig. 5(a) are smaller in Fig. 4(d) than for Fig. 4(e).

Difference (i) is most likely caused by the dependence of refraction behaviors of the electric force lines on the ZnTe layer thickness (Fig. 5(b)), which could cause the resolution difference. An inclining electric force line originating from the electrode goes upward through the 1.5 μm ZnTe layer with a small crank caused by double refractions, turns downward over the ZnTe layer, and comes back through the ZnTe layer as a negative electric field toward the grounded *n*-type Si substrate. In the 10 μm ZnTe layer (Fig. 4(e)), this turnaround behavior is much less pronounced due to the possible absence of the double refraction. In addition, the EO signals of the downward fields in the lower part of the ZnTe layer could be partly canceled by those of upward electric fields in its upper part due to their fringe geometry.

Difference (ii) is most likely due to the different vertical EO interaction lengths determined by the ZnTe layer thicknesses (Fig. 5(b)). The amplitude ratio is, however, less than the ZnTe layer thickness ratio, which has led to the sufficient imaging sensitivity of the 1.5 μm thick ZnTe plate. This sublinear sensitivity dependence on the EO plate thickness is not consistent with the super linear responsivity dependence predicted from the depolarization effect for a $\bar{4}$2m symmetry-like EO crystal^22^ and, therefore, other mechanisms are suggested. Possibly, a smaller contribution of the upper part of the 10 μm ZnTe layer to EO sensitivity gives rise to the sublinear dependence, which is ascribed to the less electric field due to its fringe distribution (Fig. 5(b)). This sublinear property should be clarified more quantitatively, for which electromagnetic field simulations should be effective, where anti-reflection (AR) and high-reflection (HR) coating layers as well as the glass platform should be taken into account. Results of ongoing numerical simulation work will be reported somewhere else.

In Fig. 4(d), something curious appears. The EO image along each of the three comb-tooth electrodes is slightly but undeniably different from the others. As for the center electrode, the *E*_*z*_ amplitude is higher and the *E*_*z*_ distribution waist is lower in the vertical position, while this is reversed for the other two electrodes. The causes for this are unclear at present, but it could be due to deformations in the comb-teeth geometry or patterns resulting from randomly-distributed damages along the line electrodes.

Finally, residual resolution-limiting factors will be discussed in two parts for further improvement. Firstly, there are four limiting factors regarding the optics as mentioned in the Result section: the diffraction limit, aberration, interference fringe, and multiple reflections. Those factors limit the resolution as follows. The present 780 nm diffraction limit of the microscope objective lens, which is the most fundamental, is 0.95 μm. The aberration caused by the 5 mm glass portion together with the interference fringe pattern effect limits the resolution to 1.6 μm as experimentally determined in the Results section while aberration caused by the ZnTe layer is negligible since its thickness is less than the glass portion by three orders of magnitudes or more although its refractive index is approximately twice as high. The interference fringe patterns of the 780 nm laser light add unnecessary undulation to EO images as in the optical 780 nm images, leading to degraded LEI resolution. The replica images in Fig. 4(b), which is caused by the multiple reflection of the 780 nm laser light at the CMOS image sensor cover glass, may have affected the profile plot in Fig. 5(a) through some coinciding shoulder features in the right hand sides of the peaks. The multiple reflections might be more troublesome in the future LEI system with an ultimate resolution although the diffraction limit, aberration, and interference fringe are dominant in the present optics.

Secondly, there are two possible resolution-limiting factors related with the EO interaction. One is the ZnTe layer thickness, which defines the sensitivity volume^23^, and its effect was discussed in part above. Preliminary simulation work performed separately suggests that the resolution is limited to approximately two thirds of the ZnTe layer thickness due to the fringe geometry of the electric field distributions. Consequently, the present 1.5 μm thickness of the ZnTe layer restricts the resolution to 1.0 μm. The effect of invasiveness on the images is unclear at present, and should be clarified in future works. The other limiting factor is the non-zero distance between the ZnTe layer bottom and the DUT surface, causing the electric fields to spread laterally before they penetrate into the ZnTe layer. Possible causes of the detachment are the HR optical coat and unintentional dust inclusion in between the surfaces. The present HR coat is approximately 1.4 μm thick, and some dust particles of several microns in outer diameter were found in optically monitoring images.

To summarize, the resolution in Fig. 4 is determined not by a single dominant factor but by multiple partial causes. Therefore, it is necessary to suppress each of these for further resolution improvement. A microscope objective lens with a larger numerical aperture (NA) value and an aberration-correction mechanism, as well as a thinner glass portion for the EO plate, could improve the optical system. In addition, the multiple reflections at the cover glass of the CMOS image sensor as well as the residual reflections in the optics should be suppressed somehow. Regarding the thicknesses of the ZnTe layer and HR coating, further reductions should be made. Avoiding dust contamination is crucial, for which a clean room environment would be necessary, while the DEI method^19^ could be effective in an alternative manner.

## Methods

### Operation principle of live electrooptic imaging

Although the details of LEI operation principles are available in our previously-published papers^1–3^, the following is a brief overview. An optical local oscillator (LO) source in Fig. 1(a) generates 780 nm laser light modulated at an LO frequency of *f*_LO_ and launched into polarization optics via a polarization maintaining optical fiber code and a collimating lens having NA of 0.19 and focal length of 60 mm. This laser light travels to a ZnTe layer where Pockels effect takes place in accordance with electric field distributions, overlaying RF phase modulation at a frequency of *f*_RF_ and resulting in spatially coherent frequency down conversion. Optical intermediate frequency (IF) components at 5 kHz thus generated within the laser beam are converted into intensity modulation at a polarization beam splitter and detected in quadrature by each of the 100 × 100 pixels on a fast CMOS image sensor, creating real-time displays of three kinds of electric field videos: magnitude, phase, and phasor images. Simultaneously, optical images of DUT illuminated by a 640 nm LED are monitored through the EO plate using an image sensor with 1600 × 1200 pixels.

### Improved microscopy optics

A finite correction optical system including an imaging lens having NA of 0.004 and focal distance of 247 mm without a focus adjusting mechanism was used in the conventional LEI system^1–3^. In the new LEI system, it was replaced with an infinity correction optical system for microscopy (Fig. 1) with fine focus adjusting mechanisms, where the magnification power of the optics was set so that approximately two pixels of the CMOS image sensor are in the diffraction limit (0.95 μm) as in usual microscopes.

The microscopy optics is schematically shown in Fig. 1(b). NA and focal distance (*d*_1_) of the microscope objective lens (Mitsutoyo M PLAN APO NR 100×) are 0.5 and 2 mm, respectively. Its rather long working distance (12 mm) suppresses the invasiveness induced by its metal housing. NA and focal distance (*d*_2_) of the imaging lens is 0.027 and 400 mm, respectively, and resultant magnification power is 200. Since an objective lens having NA of 0.14 and *d*_1_ of 40 mm is also attached to the optics together with an imaging lens having NA of 0.070 and *d*_2_ of 200 mm, the available smallest magnification power is 5. Thus, the highest optical magnification ratio in an LEI system has been renewed from the conventional (8.5)^2^ to the present (40) by a factor more than 4.7.

### Thinned electrooptic sensor plate

Figure 2 shows a (100) ZnTe EO sensor plate schematically in (a) and as a photographed with a flipped condition in (b). Its EO sensitivity is in the *z* direction. As shown in the cross-sectional side view in Fig. 2(a), it is structurally the same as the previous, where the ZnTe layer thickness was 10 μm^21^. Here, the ZnTe layer has been thinned to 1.5 μm by means of finer polishing process. HR and AR coatings for 780 nm light were made to the bottom and top, respectively. Thickness and relative permittivity ε_r_ of the HR coat are 1.4 μm and 12.2, respectively, while those of the AR coat between ZnTe and the glass platform (ε_r_ = 5.8) are 0.08 μm and 24, respectively.

### LEI resolution targets

As shown in Fig. 3,a DUT sample has been prepared as LEI resolution target. Microstrip lines (MSLs) on an *n*-type 525 μm thick (100) silicon substrate were formed by patterning a 1 μm thick Al layer on a 1 μm thick SiO_2_ layer by wet etching. The Al and SiO_2_ layers were deposited on the Si substrate by sputtering. Seven sets of interlaced interdigital electrodes were formed using the photolithography mask pattern shown in Fig. 3(a), and each of these is identified by *m* and separated by an interval *g* of 50 μm. The upper and lower bases of the comb-like patterns are at a distance *L* of 900 μm with an interlaced length *L*_IL_ of 800 μm. Each set consists of a pair of three comb teeth with line and space ranges from 3 to 12 μm. These Al lines were narrowed by 1.5 μm through side etching. For example, a comb tooth of *m* = 1 is 1.5 μm wide.

Figure 3(b) shows the Al pattern on the Si substrate die-bonded to glass epoxy PCB. The left-hand ends of the Al pattern were wire-bonded to MSLs on PCB, which originate from sub-miniature type A receptacles on the left edge of the PCB (Fig. 3(c)). Electrical signals generated by an RF synthesizer are fed to the upper receptacle, while the lower receptacle is terminated at 50 ohms.

### Data availability

There are no restrictions on the availability of materials or information.
